# Characterization of two novel colistin resistance gene *mcr-1* variants originated from *Moraxella* spp.

**DOI:** 10.3389/fmicb.2023.1153740

**Published:** 2023-05-16

**Authors:** Yongliang Che, Renjie Wu, Hongjie Li, Longbai Wang, Xuemin Wu, Qiuyong Chen, Rujing Chen, Lunjiang Zhou

**Affiliations:** ^1^Institute of Animal Husbandry and Veterinary Medicine, Fujian Academy of Agricultural Sciences, Fuzhou, China; ^2^Fujian Animal Diseases Control Technology Development Center, Fuzhou, China

**Keywords:** pig, *Moraxella*, *mcr-1* variant, colistin, resistance, *mcr-1.35*, *mcr-1.36*

## Abstract

This study aimed to characterize two novel *mcr-1* variants, *mcr-1.35* and *mcr-1.36*, which originated from *Moraxella* spp. that were isolated from diseased pigs in China. The *Moraxella* spp. carrying novel *mcr-1* variants were subjected to whole-genome sequencing (WGS) and phylogenetic analysis based on the 16S rRNA gene. The *mcr-1* variants *mcr-1.35* and *mcr-1.36* were characterized using phylogenetic analysis, a comparison of genetic environments, and protein structure prediction. The WGS indicated that two novel *mcr-1* variants were located in the chromosomes of three *Moraxella* spp. with a genetic environment of *mcr-1*-*pap2*. In addition to the novel colistin resistance genes *mcr-1.35* and *mcr-1.36*, the three *Moraxella* spp. contained other antimicrobial resistance genes, including *aac(3)-IId*, *tet*(O), *sul2*, *floR*, and *bla*_ROB-3_. A functional cloning assay indicated that either the *mcr-1.35* or *mcr-1.36* gene could confer resistance to colistin in *Escherichia coli* DH5α and JM109. The nucleotide sequences of *mcr-1.35* and *mcr-1.36* presented 95.33 and 95.33% identities, respectively, to *mcr-1.1*. The phylogenetic analysis showed that *mcr-1.35* and *mcr-1.36* were derived from *Moraxella* spp. that belonged to subclades that were different from those of the *mcr-1* variants (*mcr-1.1* to *mcr-1.34* except *mcr-1.10*) originating from Enterobacteriaceae. The deduced amino acid sequences of MCR-1.35 (MCR-1.36) showed 96.67% (96.49%), 82.59% (82.04%), 84.07% (83.52%), 55.52% (55.17%), 59.75% (59.57%), and 61.88% (61.69%) identity to MCR-1.10, MCR-2.2, MCR-6.1, MCR-LIN, MCR-OSL, and MCR-POR, respectively, that originated from *Moraxella* sp. Notably, protein structure alignment showed only a few changes in amino acid residues between MCR-1.1 and MCR-1.35, as well as between MCR-1.1 and MCR-1.36. In conclusion, this study identified *Moraxella* spp. carrying two novel *mcr-1* variants, *mcr-1.35* and *mcr-1.36*, conferring resistance to colistin, which were isolated from pig farms in China. In addition, *mcr*-like variants were observed to be located in the chromosomes of some species of *Moraxella* isolated from pig samples.

## Introduction

The overuse and misuse of antibiotics have resulted in the emergence of multidrug-resistant bacteria that pose a serious threat to people worldwide (Boucher et al., 2009; Nordmann and Poirel, 2016; Pham Thanh et al., 2016). Colistin is one of the last-line treatment options against infection by multidrug-resistant and carbapenem-resistant Gram-negative bacteria (Liu et al., 2016). However, the emergence of mobile colistin-resistant *mcr-1* genes threatens the clinical efficacy of colistin. Epidemiological studies have found the *mcr*-like genes in *Enterobacteriaceae*, *Moraxellaceae*, *Vibrionaceae*, and *Pseudomonadaceae* (Ling et al., 2017; Caselli et al., 2018; Ling et al., 2020). To date, variants from *mcr-1.1* to *mcr-1.34* and from *mcr-2* to *mcr-10* have been discovered in over 40 countries across five continents (Wang et al., 2018; Ling et al., 2020; Wang et al., 2020). With the exception of *mcr-1.10*, which was found in *Moraxella* spp., variants from *mcr-1.1* to *mcr-1.34* have been identified in various Enterobacteriaceae of different origins (AbuOun et al., 2017; Ling et al., 2020; Wang et al., 2020). Other *mcr* genes, including *mcr-5*, *mcr-8*, *mcr-9*, and *mcr-10*, have also been described in Enterobacteriaceae. Moreover, *mcr-3* and *mcr-7* were reported in *Aeromonas* spp. (Ling et al., 2020), and *mcr-4* was found in *Pseudomonas* spp. (Ling et al., 2020). Additionally, a genetic and bioinformatics analysis suggested that *Moraxella* spp. could be considered a potential source of MCR-1/2-like determinants (Kieffer et al., 2017). Moreover, several variants of the *mcr*-like genes, including *mcr-1.10*, *mcr-2.2*, and *mcr-6.1*, were first found in *Moraxella* sp. (AbuOun et al., 2017).

In addition to colistin resistance genes, *Moraxella* spp. are also known to contain ampicillin, penicillin, quinolone, macrolide, and tetracycline resistance genes, among others (Flamm et al., 2012; AbuOun et al., 2017; Krol-Turminska et al., 2020; Raveendran et al., 2020; Zhang et al., 2022). Bacteria of the genus *Moraxella* are aerobic, rod-shaped, opportunistic Gram-negative pathogens (Vela et al., 2010; Embers et al., 2011). The genus contains 18 species, including pathogens that cause infection in humans and animals (Woodbury et al., 2009; Kubota et al., 2012). For instance, *Moraxella lacunata* can cause infections in the eye and the upper respiratory tract (Woodbury et al., 2009; Mehmeti et al., 2021), while *Moraxella osloensis* and *Moraxella catarrhalis*, which are usually isolated from the human respiratory tract, can cause meningitis and pneumonia (Murphy and Parameswaran, 2009; Lee et al., 2017). Overall, *Moraxella* spp. pose a serious threat to human and animal health.

In the present study, we characterized two novel *mcr-1* variants, *mcr-1.35* and *mcr-1.36*, which originated from *Moraxella* spp. isolated from pigs with respiratory tract disease in swine farms in China.

## Materials and methods

### Isolation, purification, and identification of *Moraxella* sp.

A total of 312 nasal swabs were collected from pigs with a respiratory disease from 15 swine farms in Fujian Province, China (Supplementary Table S1). Subsequently, the samples were transferred into tryptic soy broth (TSB) (OXOID Ltd., UK). These TSB samples were then vortexed and plated onto tryptic soy agar (TSA) (OXOID Ltd., UK) supplemented with 5% defibrillated sheep blood (Zhengzhou Jiulong Biotechnology Co., Ltd., China) and 5% bovine serum (Zhejiang Tianhang Biotechnology Co., Ltd., China). A single colony was purified, after which PCR was performed to identify the 16S rRNA gene homology of the isolates using the primers 27F/1492R (Supplementary Table S2). Subsequently, the isolates identified as *Moraxella* spp. were further studied.

### PCR screening of *mcr-1* in *Moraxella* spp. and antimicrobial susceptibility testing of *mcr-1*-positive *Moraxella* sp. isolates

PCR was performed to detect the presence of *mcr-1* in *Moraxella* sp. isolates using the primer mcr-1-F/R (Supplementary Table S2). The minimum inhibitory concentrations (MICs) of *mcr-1*-positive *Moraxella* sp. against 12 antibiotics were determined using the microbroth dilution method according to the Clinical and Laboratory Standards Institute (Wayne, 2020). The *Escherichia coli* ATCC 25922 strain was used for quality control.

### Phylogenetic analysis of amino acid sequences of the mcr-like variants and the 16S rRNA gene sequences from *Moraxella* spp.

The phylogenetic analysis of the deduced amino acid sequences of the *mcr*-like variants and the 16S rRNA gene from *Moraxella* spp. was performed using MEGA 11.0 software (Tamura et al., 2021). The *mcr*-like variants included *mcr-1.35*, *mcr-1.36*, *mcr-1.10*, *mcr-2.2*, and *mcr-6.1* polymyxin resistance genes identified in *Moraxella* spp. and those identified in *M. osloensis*, *Moraxella lincolnii*, *and Moraxella porci* (*mcr*_OSL_, *mcr*_LIN,_ and *mcr*_POR_, respectively) (Kieffer et al., 2017). In addition to the 16S rRNA genes of *mcr-1*-positive *Moraxella* spp. isolated in this study, other 16S rRNA genes from different *Moraxella* species were obtained from the NCBI database.

### Phylogenetic analysis of all *mcr-1* gene variants

The phylogenetic analysis of the complete sequences of *mcr-1.35* and *mcr-1.36* found in *Moraxella* spp., as well as the complete sequences of *mcr-1.1* to *mcr-1.34* registered in GenBank, were performed using MEGA 11.0 software (Supplementary Table S3; Tamura et al., 2021).

### Whole-genome sequencing analysis

The DNA of three *Moraxella* spp. was extracted using a bacterial DNA extraction kit (OMEGA, USA). The whole genomes of these *Moraxella* sp. were sequenced using the Illumina Hiseq and Oxford Nanopore MinIon platforms, and complete sequences were obtained by hybrid assembly using Unicycler version 0.5.0 (Wick et al., 2017). Functional element prediction was conducted using Prokka (Seemann, 2014). The sequences were annotated by comparing the predicted gene sequences with functional databases such as RefSeq (O'Leary et al., 2016) and CARD (Alcock et al., 2020) with BLAST+. The genetic context of *mcr-1* was analyzed using Easyfig (Sullivan et al., 2011).

### Functional cloning of novel *mcr-1* variants

A 1,623-bp DNA fragment of *mcr-1.35* and *mcr-1.36* was amplified from *Moraxella* spp. FZFQ2102, FZLJ2107, and FZLJ2109 using the primer mcr1L-F/R (Supplementary Table S2) and then cloned into pUC19 to obtain pUC19*-mcr-1.35* and pUC19*-mcr-1.3*6. The vector was transformed into *E. coli* DH5α and *E. coli* JM109. The MICs of polymyxin B against DH5α and JM109 harboring pUC19-*mcr-1.35*, pUC19*-mcr-1.36*, and pUC19 were determined by the microbroth dilution method.

### Protein structure analysis of MCR-1 variants from *Moraxella* sp.

The structure of lipooligosaccharide phosphoethanolamine transferase A (*EptA*) (PDB accession number 5FGN) was obtained from the Protein Data Bank.^1^ The structures of MCR-1 variants were generated according to their amino acid sequences using the comparative protein-modeling SWISS-MODEL server (Waterhouse et al., 2018). These structures were then analyzed and visualized using PyMOL software.

### Nucleotide sequence accession numbers

The complete nucleotide sequences of *Moraxella* spp. FZFQ2102, FZLJ2107, and FZLJ2109 were deposited in GenBank under the accession numbers CP099960, CP101111, and CP101112, respectively.

## Results

### Identification of *mcr-1*-positive *Moraxella* sp.

In total, 58 isolates that belonged to *Moraxellae* were isolated and identified from the 312 samples obtained from 15 swine farms in Fujian Province, China (Supplementary Table S1). Of these 58 isolates, three were identified as positive for *mcr-1* and were named FZFQ2102, FZLJ2107, and FZLJ2109, respectively (Table 1).

**Table 1:** Characterization of the *mcr-1*-carrying *Moraxella* spp. FZFQ2102, FZLJ2107, and FZLJ2109.

Isolates	MICs of polymyxin B (μg/mL)	Other resistance phenotypes	Chromosome size (bp)	Resistance genes	*mcr-1* genetic environment
FZFQ2102	8	CHL, FFC, GEN DOX, CTX, CIP, SMZ, AZM, PEN	2,336,058	*aac(3)-IId*, *aac(3)-IV*, *aph(2″)-If*, *aph(3′)-Ia*, *aph(3″)-Ib*, *aph(6)-Id*, *erm*(T), *floR*,***mcr-1.35*,** *bla*_ROB-3_, *bla*_ROB-9_, *sul2*, *tet*(H), *tet*(O)	*mcr-1-pap-2*
FZLJ2107	8	CHL, FFC, GEN, DOX, CTX, CIP, SMZ, PEN	2,422,986	*aadA8b*, *aph(2″)-If*, *aph(3′)-Ia*, *aph(3″)-Ib*, *aph(6)-Id*, *bla*_CARB-50_, *floR*, ***mcr-1.36***, *bla*_ROB-9_, *sul2*, *tet*(H), *tet*(O)	*mcr-1-pap-2*
FZLJ2109	8	CHL, FFC, GEN DOX, CTX, CIP, SMZ, PEN	2,424,775	*aadA8b*, *aph(2″)-If*, *aph(3′)-Ia*, *aph(3″)-Ib*, *aph(6)-Id*, *bla*_CARB-50_, *floR*, ***mcr-1.36***, *bla*_ROB-9_, *sul2*, *tet*(H), *tet*(O)	*mcr-1-pap-2*

CHL, chloramphenicol; FFC, florfenicol; GEN, gentamicin; DOX, doxycycline; SMZ, sulfamethoxazole; CTX, cefotaxime; CIP, ciprofloxacin; AZM, azithromycin; PEN, penicillin. Bold respresents the colistin resistance gene.

**Figure 1:**
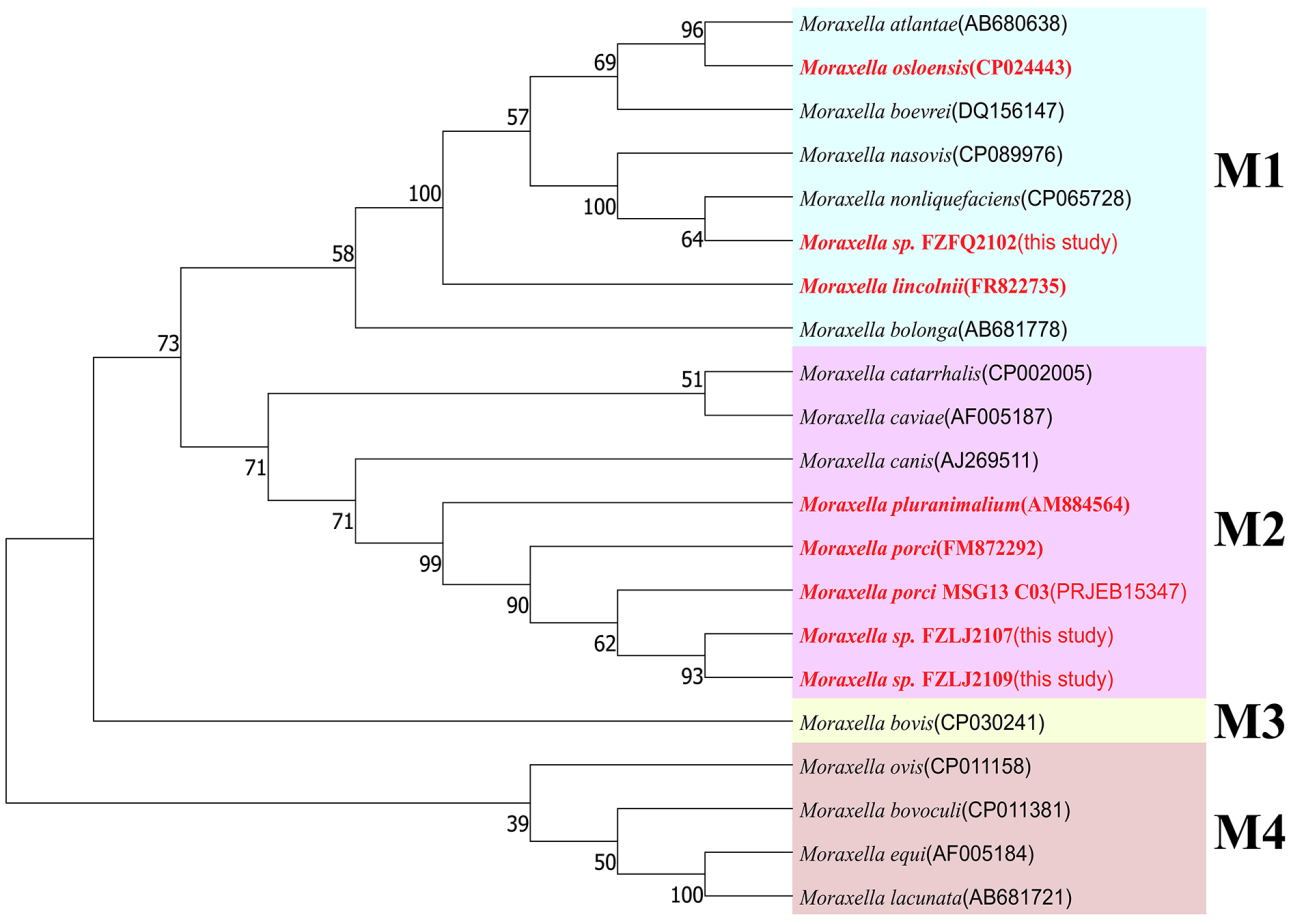
Phylogenetic tree of different *Moraxella* species based on 16S rRNA. The 16S rRNA genes of the three *mcr-1*-positive *Moraxella* spp. isolated in this study were compared with those of *Moraxella* spp. from the NCBI database. The neighbor-joining phylogeny was constructed using MEGA 11.0 with the 16S rRNA gene sequence of *Moraxella* spp. with the *mcr* gene (in red) and without the *mcr* gene (in black). Branch labels correspond to bootstrap support percentages out of 1,000 replicates. The tree is divided into four groups: M1 (light blue area), M2 (pink area), M3 (light yellow area), and M4 (light orange area).

Due to FZFQ2102, FZLJ2107, and FZLJ2109 being identified as *Moraxella* sp. by PCR, the phylogenetic analysis was performed using the 16S rRNA genes of the *Moraxella* sp. obtained in this study and those in the NCBI database. According to 16S rRNA sequence identity, the phylogenetic tree of *Moraxella* sp. was divided into four groups: M1, M2, M3, and M4. The *Moraxella* sp. FZFQ2102 shared a close phylogenetic relationship with *Moraxella nonliquefaciens* and *Moraxella nasovis*. Moreover, the *Moraxella* spp. FZLJ2107 and FZLJ2109 shared a close phylogenetic relationship with *M. porci* and *Moraxella pluranimalium* (Figure 1).

### Two novel colistin resistance gene *mcr-1* variants in *Moraxella* sp.

**Figure 2:**
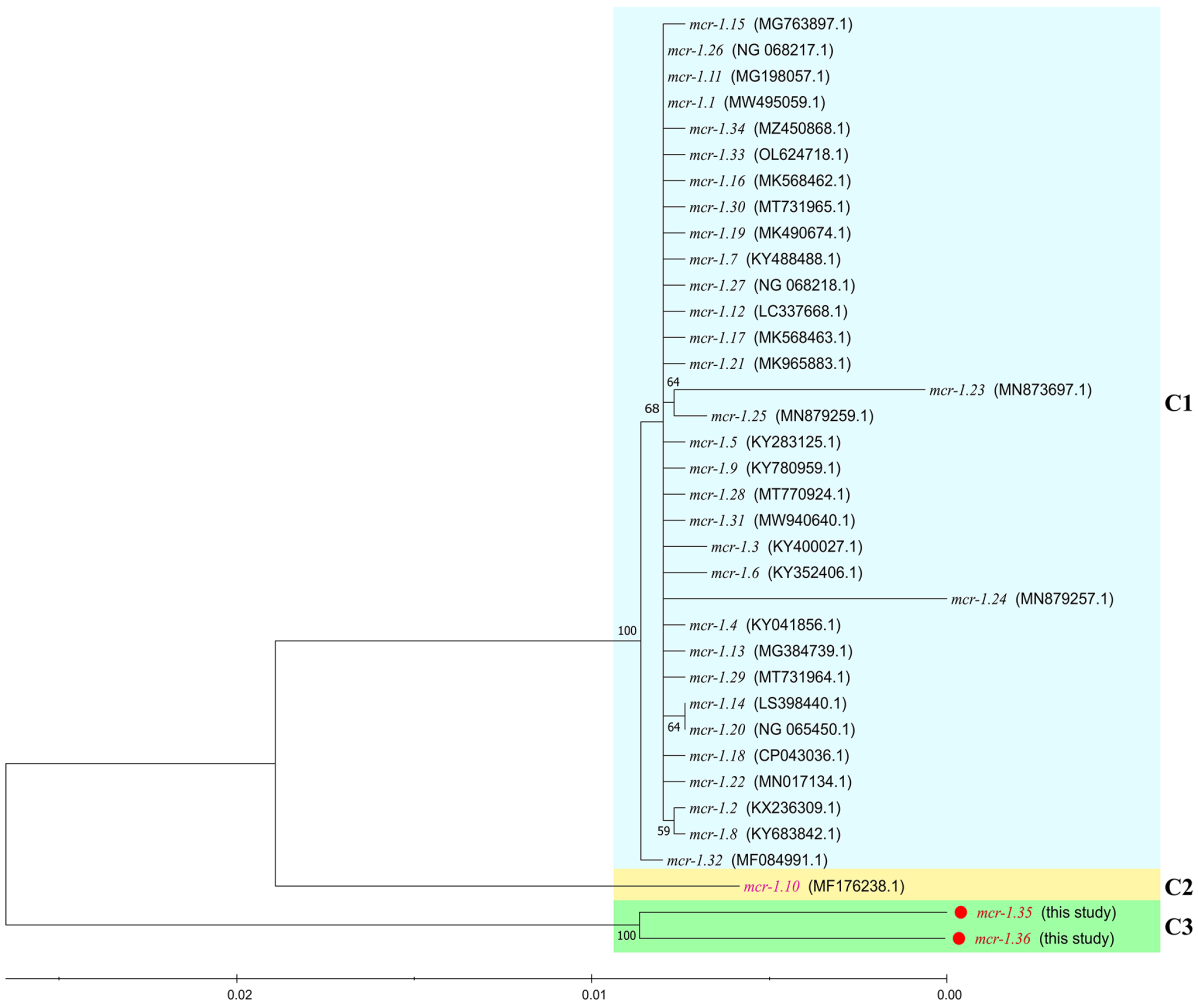
Comparison between *mcr-1.35* and *mcr-1.36* and all previously described *mcr-1* subtypes, based on nucleotide sequences. The neighbor-joining phylogeny was constructed using MEGA 11.0 with the nucleotide sequences of the novel colistin-resistance genes *mcr-1.35* and *mcr-1.36* (in red) and all previously described the *mcr-1* gene subtypes (*mcr-1.1* to *mcr-1.34* [in black and pink]). Branch labels correspond to bootstrap support percentages out of 1,000 replicates. The tree is divided into three groups: C1, which contains the *mcr-1* gene subtypes from other bacterial species (light blue area), C2, which contains the *mcr-1.10* gene from *Moraxella* spp. (light yellow area), and C3, which contains the *mcr-1.35* and *mcr-1.36* genes from *Moraxella* spp. (green area).

Between 2015 and 2022, an increasing number of *mcr*-like variants were found in *Moraxella* sp. (AbuOun et al., 2017; Kieffer et al., 2017). The three *mcr-1*-positive *Moraxella* sp. isolated in this study demonstrated multidrug resistance, including resistance to polymyxin B (MIC = 8 μg/mL) (Table 1). Sequence analysis indicated that the three *Moraxella* sp. carried two *mcr-1* variants. BLASTn results showed that the nucleotide sequence homology of the two *mcr-1* variants with *mcr-1* was 95.33% (Table 2). Furthermore, the phylogenetic analysis suggested that the nucleotide sequence identities of the two *mcr-1* variants from *Moraxella* sp. FZFQ2102, FZLJ2107, and FZLJ2109 were distinct from those of *mcr-1.1*–*mcr-1.34* (Figure 2). Therefore, the two novel *mcr-1* variants were named *mcr-1.35* and *mcr-1.36*.

**Table 2:** Pairwise comparisons of *mcr-1.1* and *mcr*-like variants in *Moraxella* sp. based on nucleotide and amino acid sequence identity (%).

	*mcr-1.1*	*mcr-1.10*	*mcr-1.35*	*mcr-1.36*	*mcr-2.2*	*mcr-6.1*	*mcr*_LIN_	*mcr*_OSL_	*mcr*_POR_
*mcr-1.1*	100	97.60	95.33	95.33	77.49	78.07	57.65	62.32	63.88
*mcr-1.10*	98.71	100	95.02	95.20	77.06	77.33	57.99	61.91	63.57
*mcr-1.35*	97.23	96.67	100	98.27	78.62	80.18	57.88	62.68	63.14
*mcr-1.36*	96.67	96.49	99.44	100	78.13	79.69	58.11	62.74	63.50
*mcr-2.2*	81.52	81.15	82.59	82.04	100	85.16	59.81	63.97	64.31
*mcr-6.1*	82.44	81.89	84.07	83.52	87.73	100	58.28	63.00	63.18
*mcr*_LIN_	55.34	55.34	55.52	55.17	55.61	55.96	100	59.64	60.11
*mcr*_OSL_	59.04	59.57	59.75	59.57	60.21	61.46	54.97	100	60.76
*mcr*_POR_	62.13	61.76	61.88	61.69	61.44	62.66	54.30	59.33	100

Green and blue shading represent comparisons of nucleotide and amino acid sequence identity, respectively.

### Characterization of the three *mcr-1*-positive *Moraxella* sp.

Whole-genome sequencing (WGS) suggested that the three *Moraxella* sp., FZFQ2102, FZLJ2107, and FZLJ2109, have 2,336,058-bp, 2,422,986-bp, and 2,424,775-bp chromosomes, respectively, and no plasmids (Table 1). WGS also showed that the three *Moraxella* sp. possessed multiple drug-resistance genes. The *Moraxella* sp. FZFQ2102 contained the colistin resistance gene *mcr-1.35*; the aminoglycoside resistance genes *aac(3)-IId*, *aac(3)-IV*, *aph(2″)-If*, *aph(3′)-Ia*, *aph(3″)-Ib*, and *aph(6)-Id*; the macrolide resistance gene *erm*T; the tetracycline resistance genes *tet*(H) and *tet*(O); the sulfonamide resistance gene *sul2*; the phenicol resistance gene *floR*; and the β-lactamase resistance genes *bla*_ROB-3_ and *bla*_ROB-9_. Moreover, the *Moraxella* sp. FZLJ2107 and FZLJ2109 contained the colistin resistance gene *mcr-1.36*; the aminoglycoside resistance genes *aadA8b*, *aph(2″)-If*, *aph(3′)-Ia*, *aph(3″)-Ib*, and *aph(6)-Id*; the tetracycline resistance genes *tet*(H) and *tet*(O); the sulfonamide resistance gene *sul2*; the phenicol resistance gene *floR*; and the β-lactamase resistance genes *bla*_ROB-9_ and bla_CARB-50_ (Table 1).

**Figure 3:**
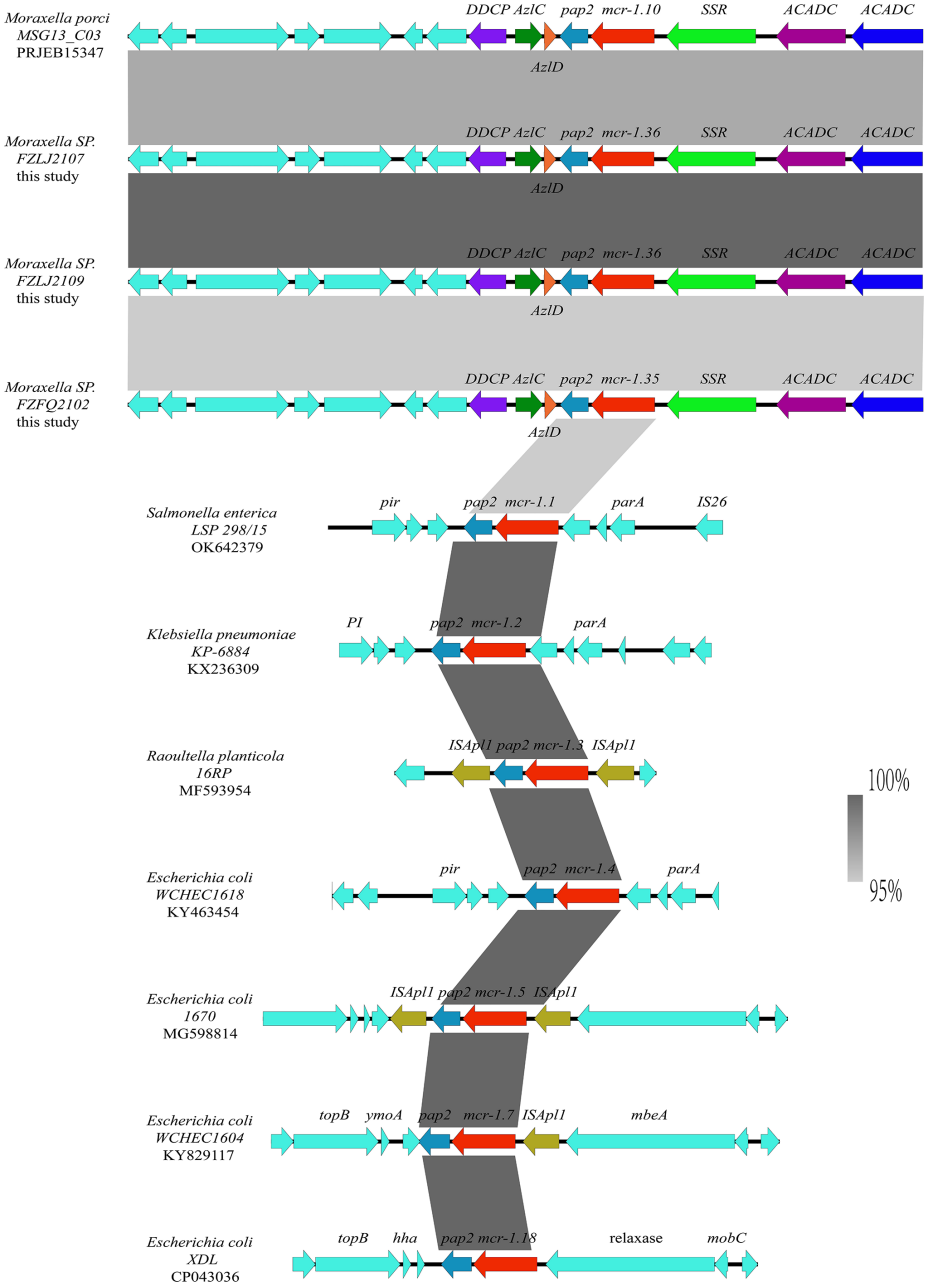
Comparison between the genetic context of the *mcr-1* subtypes from *Moraxella* spp. and those from other bacterial species. The extents and directions of genes are shown by arrows labeled with gene names, with the *mcr-1* subtypes represented by red arrows and the *pap2* gene represented by a light blue arrow. Other flanking genes have been annotated. The shadow parallelograms between each sequence denote sequence identity.

The analysis of the context of the *mcr-1* variants suggested that their flanking regions were highly similar among the *Moraxella* sp. The *mcr* genes in the *Moraxella* sp. were downstream of a site-specific recombinase gene (*ssr*) and upstream of the *pap-2* gene. However, the genetic environment of *mcr-1* variants in other *mcr-1*-carrying bacteria differed from that of the *Moraxella* sp. Interestingly, *mcr-1* variants had a common genetic unit: *mcr-1-pap2* (Figure 3).

### Functional confirmation of *mcr-1.35* and *mcr-1.36*

BLASTn analysis confirmed that two 1,623-bp open reading frames (ORFs) encoded the putative phosphoethanolamine transferase, which showed high nucleotide sequence identity to the original *mcr-1.1* gene. The amino acid sequences of MCR-1.35 and MCR-1.36 showed 97.23 and 96.67% identity, respectively, to that of MCR-1.1 (Table 2). To confirm the function of the putative phosphoethanolamine transferase, the recombinant vectors pUC19-*mcr-1.35* and pUC19-*mcr-1.36* were constructed and subsequently transformed into *E. coli* DH5α and JM109. Compared with *E. coli* DH5α and JM109 carrying pUC19 alone, the expression of these ORFs in *E. coli* DH5α and JM109 led to 4-fold increases in the MIC of polymyxin B, which further identified the function of the *mcr-1.35* and *mcr-1.36* genes (Table 3).

**Table 3:** MICs of polymyxin B for the constructed strains.

Species	MICs (μg/mL)
	Polymyxin B
*E. coli* DH5α + pUC19-*mcr-1.35*	2
*E. coli* DH5α + pUC19-*mcr-1.36*	2
*E. coli* DH5α + pUC19	0.5
*E. coli* JM109 + pUC19-*mcr-1.35*	2
*E. coli* JM109 + pUC19-*mcr-1.36*	2
*E. coli* JM109 + pUC19	0.5
*E. coli* 25,922	0.25

### Analysis of *mcr*-like variants from *Moraxella* sp.

BLASTn results showed that *mcr-1.35* and *mcr-1.36* exhibited more than 95% nucleotide sequence identity to *mcr-1.10*, indicating a close phylogenetic relationship. *Mcr-1.35* and *mcr-1.36* also exhibited 78.62 and 78.13% nucleotide sequence identity to *mcr-2.2* and 80.18 and 79.69% nucleotide sequence identity to *mcr-6.1*, respectively. Furthermore, *mcr-1.35* (*mcr-1.36*) showed 57.88% (58.11%), 62.68% (62.74%), and 63.14% (63.50%) nucleotide sequence identity to the *mcr*_LIN_, *mcr*_OSL,_ and *mcr*_POR_ genes found in *M. osloensis*, *M. porci*, and *M. lincolnii*, respectively. Additionally, *mcr-1.35* exhibited 98.27% nucleotide sequence identity to *mcr-1.36* in this study (Table 2).

**Figure 4:**
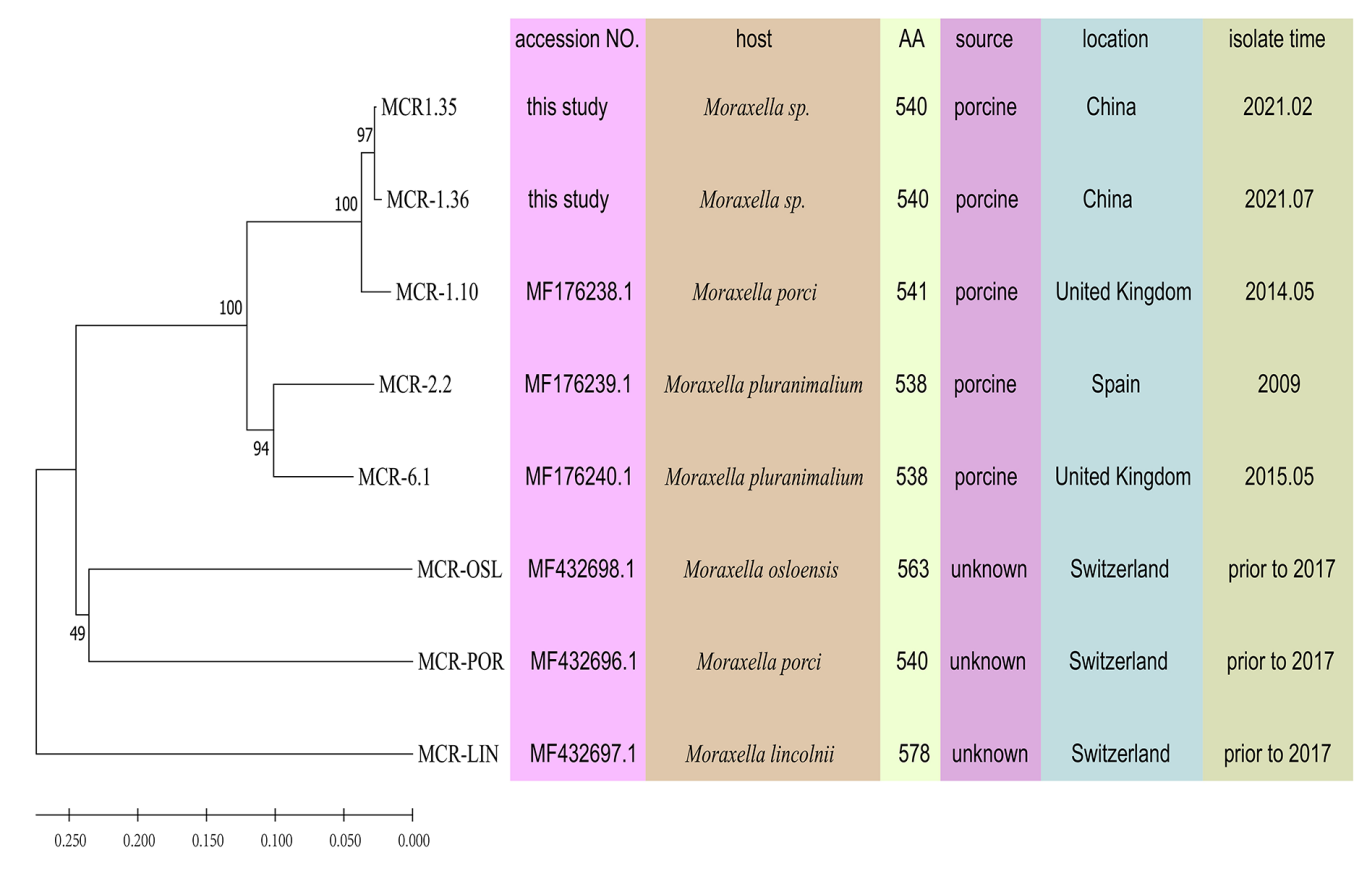
Phylogeny of MCR-like variants from *Moraxella* spp. determined based on amino acid sequences using MEGA 11.0. MCR-OSL, MCR-POR, and MCR-LIN are polymyxin resistance determinants that were identified in *M. osloensis*, *M. porci* and *M. lincolnii*, respectively. Some parameters relevant to the MCR proteins are presented, including bacterial host, the number of amino acids, isolate source, isolate location, and isolate time.

Compared with other phosphoethanolamine transferases in *Moraxella* sp., MCR-1.35, and MCR-1.36 aligned closely with an MCR-1.10 found in *M. porci* isolated from a pig in Great Britain (> 96%). MCR-1.35 and MCR-1.36 also exhibited 82.59 and 82.04% amino acid identity to MCR-2.2 and 84.07 and 83.52% amino acid identity to MCR-6.1, found in *Moraxella pluranimalium* isolated from pigs in Europe, respectively. In addition, MCR-1.35 (MCR-1.36) showed 55.52% (55.17%), 59.75% (59.57%), and 61.88% (61.69%) amino acid sequence identity to the MCR-OSL, MCR-POR, and MCR-LIN proteins found in *M. osloensis*, *M. porci*, and *M. lincolnii*, respectively (Table 2; Figure 4).

### Structure prediction and amino acid sequence analysis of MCR-1 variants from *Moraxella* sp.

**Figure 5:**
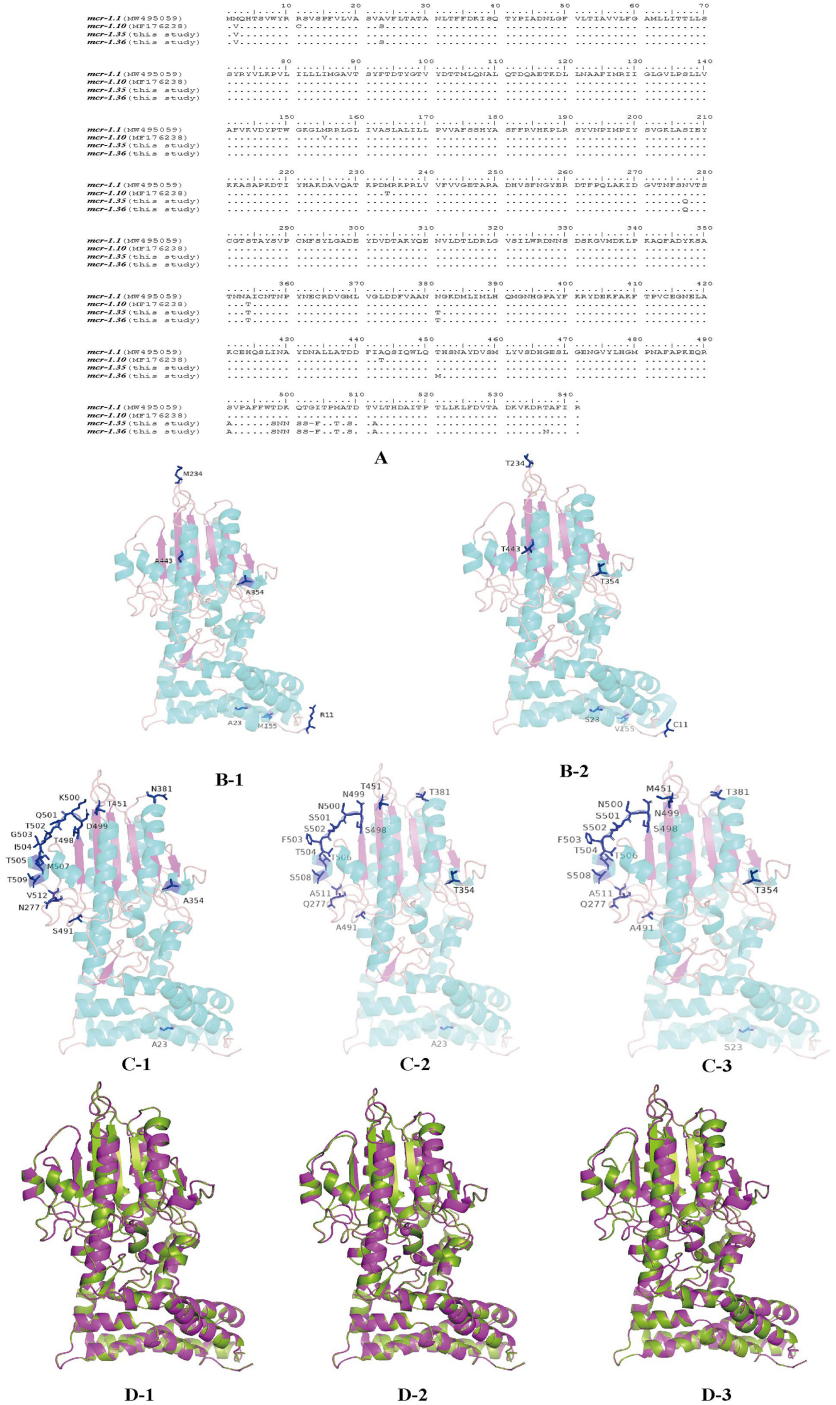
Structures of MCR-1 variants (MCR-1.1, MCR-1.10, MCR-1.35, and MCR-1.36) **(A)** Amino acid sequences of MCR-1.10, MCR-1.35, and MCR-1.36 compared with MCR-1.1. Amino acid residues are depicted in black, and the same amino acids are represented by dots in the alignment. **(B)** Structural models comparison of the MCR-1.1 protein (B-1) and MCR-1.10 (B-2) from *Moraxella* spp. based on lipooligosaccharide phosphoethanolamine transferase EptA. **(C)** Structural models comparison of the MCR-1.1 protein (C-1), and MCR-1.35 (C-2), MCR-1.36 (C-3) from Moraxella spp. based on lipooligosaccharide phosphoethanolamine transferase EptA. Models were constructed using the SWISS-MODEL server, and structures were viewed and edited using PyMOL2.5.4. Mutated amino acids are shown in the structural model. **(D)** Comparison between the structure of MCR-1.1 (in green) and that of MCR-1.10 (D-1), MCR-1.35 (D-2) and MCR-1.36 (D-3) (in pink).

MCR-1 variants from *Moraxella* sp. were aligned using BioEdit (Figure 5A). When aligned with MCR-1.1, MCR-1.10 presented six amino acid mutations at residues R11C, A23S, M155V, M234T, A354T, and A443T. Four residues, S^23^, V^155^, T^354^, and T^443^, were located in the α-helix of the secondary structure, while residues C^11^ and T^234^ were located in the random coil. However, compared with those of MCR-1.1, the amino acid sequences of MCR-1.35 exhibited changes at residues Q^277^, T^354^, T^381^, A^491^, S^498^, N^499^, N^500^, S^501^, S^502^, 503 deletion, F^503^, T^506^, S^508^, and A^511^. Three residues, T^354^, T^506^, and S^508^, were located in the α-helix on the secondary structure, while all other residues were located in the random coil. In addition, amino acid sequences of MCR-1.36 presented changes at residues S^23^, Q^277^, T^354^, T^381^, M^451^, A^491^, S^498^, N^499^, N^500^, S^501^, S^502^, 503 deletion, F^503^, T^506^, S^508^, and A^511^. In addition, the four residues S^23^, T^354^, T^506^, and S^508^ were located in the α-helix on the secondary structure, with all other residues located in the random coil (Figures 5C, D). Notably, protein structure alignment revealed only a few differences between MCR-1.1 and MCR-1.10, MCR-1.1 and MCR-1.35, and MCR-1.1 and MCR-1.36 (Figure 5D).

## Discussion

*Moraxella* spp. have been identified as potential sources of MCR-like polymyxin resistance determinants (Kieffer et al., 2017; Poirel et al., 2017). One study identified the *mcr*-like genes, including *mcr-1.10* (*M. porci*), *mcr-2.2* (*M. pluranimalium*), and *mcr-6.1* (*M. pluranimalium*), in *Moraxella* spp. isolated from the cecal contents of healthy pigs on farms in Great Britain (AbuOun et al., 2017). Furthermore, the proteins MCR-POR (*M. porci*), MCR-OSL (*M. osloensis*), and MCR-LIN (*M. lincolnii*) in *Moraxella* spp. shared high amino acid identities with MCR-1/2-like (Kieffer et al., 2017). In the present study, the novel *mcr-1* variants *mcr-1.35* and *mcr-1.36* were identified in *Moraxella* spp. recovered from the nasal swabs of pigs with respiratory diseases in farms in China. Notably, the emergence of different *mcr*-like variants in various *Moraxella* spp. indicates that the *mcr*-like variants were stored in the chromosomes of some *Moraxella* species.

Three *Moraxella* spp. containing novel *mcr-1* variants showed resistance to polymyxin (Table 1). Additionally, the MIC of colistin for *Moraxella* spp. containing *mcr-1.10*, *mcr-2.2*, and *mcr-6.1* ranged from 1 to 2 μg/mL (AbuOun et al., 2017), while the MIC of colistin for EptA-containing *Moraxella* spp. ranged from 2 to 64 μg/mL (AbuOun et al., 2017; Kieffer et al., 2017). Moreover, *mcr-1.35*, *mcr-1.36*, *mcr-2.2*, *mcr*_osl_, *mcr*_lin,_ and *mcr*_por_ conferred resistance to polymyxin in *E. coli* (Kieffer et al., 2017; Poirel et al., 2017). In addition to colistin resistance genes (*mcr-1.10*, *mcr-1.35*, *mcr-1.36*, *mcr-2.2*, and *mcr-6.1*), *Moraxella* spp. also carried genes for β-lactam resistance (*bla*_BRO-1_ and *bla*_BRO-2_) aminoglycoside resistance (*aac(3)-IId*, *aac(3)-IV*, *aph(2″)-If*, *aph(3′)-Ia*, *aph(3″)-Ib*, and *aph(6)-Id*); tetracycline resistance [*tet*(B), *tet*(D), *tet*(L), and *tet*(O)]; chloramphenicol resistance (*floR*), sulfonamide resistance (*sul2*), and macrolide resistance (*erm*T) (Flamm et al., 2012; AbuOun et al., 2017; Krol-Turminska et al., 2020; Raveendran et al., 2020; Zhang et al., 2022). In recent studies, *Moraxella* spp. from human samples have proven to be mainly resistant to β-lactam, tetracycline, and macrolide (Flamm et al., 2012; Krol-Turminska et al., 2020; Raveendran et al., 2020; Zhang et al., 2022). The three *Moraxella* spp. from animal samples collected in this study showed multidrug resistance, indicating that *Moraxella* spp. pose potential threats to human and animal health.

The phylogenetic analysis showed that the *mcr-1* variants (*mcr-1.10*, *mcr-1.35*, and *mcr-1.36*) that originated from *Moraxella* spp. belonged to subclades (C2 and C3 group) distinct from that of the *mcr-1* variants originating from *Enterobacteriaceae* (C1 group) (Figure 2). The phylogenetic relationships between *mcr-1.35*, *mcr-1.36*, and *mcr-1.1* differed, but the products of these genes are known to be responsible for polymyxin resistance (Liu et al., 2016). The comparisons of the nucleotide sequences showed that the context of the *mcr-1* variants was similar among *Moraxella* spp. but differed from the genetic environment of the *mcr-1* variants in other bacteria. These results indicate that *mcr-1-pap2* acts as the genetic unit of the *mcr-1* variants in *Moraxella* spp. (AbuOun et al., 2017; Poirel et al., 2017).

Compared with that of MCR-1.1, the amino acid sequences of MCR-1.35 and MCR-1.36 exhibited mutations located at the α-helix and random coil on the secondary structure. Previous research has suggested that the eight active sites (E246, T285, K333, H395, D465, H466, E468, and H478) located on the β-sheet of the protein’s secondary structure are essential for the activity of MCR-1 (Hu et al., 2016; Ma et al., 2016; Stojanoski et al., 2016; Hinchliffe et al., 2017). Notably, these β-sheet mutations were highly conserved in MCR-1.35 and MCR-1.36 ORFs (Carroll et al., 2019; Wang et al., 2020), indicating that the mutations of MCR-1.35 and MCR-1.36 have no influence on their polymyxin resistance activity. Furthermore, protein structure alignment indicated that the structures of MCR-1.35, MCR-1.36, and MCR-1.1 did not differ significantly.

In conclusion, this study identified the *Moraxella* spp. carrying two novel *mcr-1* variants, *mcr-1.35* and *mcr-1.36*, conferring resistance to colistin, which were isolated from pig farms in China. *Moraxella* spp. were considered potential sources of MCR-like determinants, and the *mcr*-like variants were observed to be located in the chromosomes of some *Moraxella* species isolated from pig samples. Therefore, further research on the *mcr*-like genes in *Moraxella* spp. may help us understand the evolution and spread of the *mcr*-like genes.

## Data availability statement

The datasets presented in this study can be found in online repositories. The names of the repository/repositories and accession number(s) can be found in the article/Supplementary material.

## Author contributions

LZ and YC designed the experiments. YC, RW, and HL performed the experiments. LW and XW prepared the tables and figures. YC, RW, QC, and RC prepared the manuscript. All authors read and approved the final version of the manuscript.

## Funding

This study was supported by the National Nature Science Foundation of China (No. 31872502), basic scientific research projects of provincial public welfare scientific research institutions (Fujian Province), China (No. 2022R10260013), and the key project of the Fujian “5511” Collaborative Innovation Project, China (No. XTCXGC2021008).

## Conflict of interest

The authors declare that the research was conducted in the absence of any commercial or financial relationships that could be construed as a potential conflict of interest.

## Publisher’s note

All claims expressed in this article are solely those of the authors and do not necessarily represent those of their affiliated organizations, or those of the publisher, the editors and the reviewers. Any product that may be evaluated in this article, or claim that may be made by its manufacturer, is not guaranteed or endorsed by the publisher.

## Footnotes

^1^http://www.rcsb.org/pdb/
