# Supplementary material for: Characterization of two novel colistin resistance gene mcr-1 variants originated from Moraxella spp
Source: Front Microbiol. 2023 May 16;14:1153740. doi: 10.3389/fmicb.2023.1153740 (PMC10228737; doi:10.3389/fmicb.2023.1153740)
Supplement: Supplementary file 1 [file Data_Sheet_1.PDF]

Table S1 Sample information of 58 strains of *Moraxella sp.*

Isolation pig farm	Isolation location	Isolation strains	Isolation time
A	Fuqing City	3	2/2021
B	Fuqing City	4	10/2021
C	Lianjiang County	6	7/2021
D	Lianjiang County	4	9/2021
E	Minhou County	2	5/2020
F	Liancheng County	11	7/2020
G	Qingliu County	5	10/2020
H	Longyan City	7	12/2021
I	Nanjing County	5	10/2020
J	Nanjing County	0	11/2021
K	Zhangpu County	2	1/2022
L	Changtai County	3	2/2022
M	Xiapu County	0	11/2020
N	Fuding County	5	3/2022
O	Putian City	1	8/2021

Note: A-O represents different fifteen pig farms. Isolation locations are from Fujian Province, China. *Moraxella sp.* strain FZFQ2102 was isolated from pig farm A. *Moraxella sp.* strain FZLJ2107 was isolated from pig farm C. *Moraxella sp.* FZLJ2109 was islated from pig farm D.

Table S2 The sequence of primers used in this study

Target segment	Primers	Sequences (5' to 3')	Reference
16s rRNA	27F	AGAGTTTGATCATGGCTCAG	(1)
16s rRNA	1492R	GGTACCTTGTTACGACTT	(1)
mcr-1	mcr-1-F	AGTCCGTTTGTTCTTGTGGC	(2)
mcr-1	mcr-1-R	AGATCCTTGGTCTCGGCTTG	(2)
mcr-1	mcr1L-F	CCCAAGCTTATGATGCAGCATACTTCTG	This study
mcr-1	mcr1L-R	CTAGTCTAGATCAGCGGATGAATGCGGTG	This study

Table S3 List of used *mcr*-like variants in this study

Variant	Accession number	Homology(%)	Host strain	Source	Country
mcr-1.1	KP347127.1	100	*Escherichia coli*	Pig	China
mcr-1.2	KX236309.1	99.94	*Klebsiella pneumoniae*	Human	Italy
mcr-1.3	KY400027.1	99.88	*Escherichia coli*	Mink	China
mcr-1.4	KY041856.1	99.94	*Escherichia coli*	Sewage	China
mcr-1.5	KY283125.1	99.94	*Escherichia coli*	Human	Argentina
mcr-1.6	KY352406.1	99.88	*Salmonella enterica*	Human	China
mcr-1.7	KY488488.1	99.94	*Escherichia coli*	Sewage	China
mcr-1.8	KY683842.1	99.94	*Escherichia coli*	Poultry	Brunei
mcr-1.9	KY780959.1	99.94	*Escherichia coli*	Pig	Portugal
mcr-1.10	MF176238.1	97.60	*Moraxella sp.*	Pig	UK
mcr-1.11	MG198057.1	99.82	*Escherichia coli*	Pig	UK
mcr-1.12	LC337668.1	99.94	*Escherichia coli*	Pork	Japan
mcr-1.13	MG384739.1	99.94	*Escherichia coli*	Turkey meat	Germany
mcr-1.14	LS398440.1	99.94	*Escherichia coli*	Pig	UK
mcr-1.15	MG763897.1	99.75	*Klebsiella pneumoniae*	Chicken	China
mcr-1.16	MK568462.1	99.94	*Escherichia coli*	Chicken	China
mcr-1.17	MK568463.1	99.94	*Escherichia coli*	Cow	China
mcr-1.18	CP043036.1	99.94	*Escherichia coli*	Human	China
mcr-1.19	MK490674.1	99.94	*Salmonella enterica*	Egg	China
mcr-1.20	NG065450.1	99.94	*Escherichia coli*	Pig	UK
mcr-1.21	MK965883.1	99.94	*Escherichia coli*	Human	China
mcr-1.22	MN017134.1	99.94	*Escherichia coli*	Broiler chicken	Nigeria
mcr-1.23	MN873697.1	99.20	*Salmonella enterica*	Poultry	Bangladesh
mcr-1.24	MN879257.1	99.20	*Escherichia coli*	Poultry	Bangladesh
mcr-1.25	MN879259.1	99.88	*Escherichia coli*	Poultry	Bangladesh
mcr-1.26	NG068217.1	99.82	*Escherichia coli*	Human	Germany
mcr-1.27	NG068218.1	99.94	*Escherichia coli*	Human	Germany

mcr-1.28	MT770924.1	99.94	*Escherichia coli*	Human	China
mcr-1.29	MT731964.1	99.94	Uncultured bacterium	Environment	China
mcr-1.30	MT731965.1	99.94	Uncultured bacterium	Swine	China
mcr-1.31	MW940640.1	99.94	*Escherichia coli*	Human	Italy
mcr-1.32	MF084991.1	99.88	*Escherichia coli*	Sputum	Spain
mcr-1.33	OL624718.1	99.94	*Escherichia coli*	Human	China
mcr-1.34	MZ450868.1	99.94	*Escherichia coli*	Human	China
mcr-1.35	this study	95.33	*Moraxella sp.*	Pig	China
mcr-1.36	this study	95.33	*Moraxella sp.*	Pig	China
mcr-2.1	LT598652.1		*Escherichia coli*	Pig	Belgium
mcr-2.2	MF176239.1		*Moraxella sp.*	Pig	Spain
mcr-3.1	CP042645.1		*Escherichia coli*	Pig	China
mcr-4.1	MG459156.1		*Escherichia coli*	Pig	Germany
mcr-5.1	AP023312.1		*Salmonella enterica*	Bovine	Japan
mcr-6.1	MF176240.1		*Moraxella sp.*	Pig	UK
mcr-7.1	MG267386.1		*Klebsiella pneumoniae*	Chicken	China
mcr-8.1	MT070402.1		*Klebsiella pneumoniae*	human	Nigeria
mcr-9.1	AP022519.1		*Enterobacter cloacae*	human	Japan
mcr-10.1	MN179494.1		*Enterobacter roggenkampii*	human	China

References

1. Zhu F, Chen L, Chen H. 2013. [Identification of pathogenic microorganism by sequencing 16S rRNA gene]. Zhong Nan Da Xue Xue Bao Yi Xue Ban 38:1035-41.

2. Liu YY, Wang Y, Walsh TR, Yi LX, Zhang R, Spencer J, Doi Y, Tian G, Dong B, Huang X, Yu LF, Gu D, Ren H, Chen X, Lv L, He D, Zhou H, Liang Z, Liu JH, Shen J. 2016. Emergence of plasmid-mediated colistin resistance mechanism MCR-1 in animals and human beings in China: a microbiological and molecular biological study. Lancet Infect Dis 16:161-8.
